# Research on the psychologically restorative effects of campus common spaces from the perspective of health

**DOI:** 10.3389/fpubh.2023.1131180

**Published:** 2023-04-13

**Authors:** Weihong Guo, Hongyan Wen, Xiao Liu

**Affiliations:** ^1^School of Architecture, South China University of Technology, Guangzhou, China; ^2^State Key Laboratory of Subtropical Building Science, South China University of Technology, Guangzhou, China; ^3^Architectural Design and Research Institute Co., Ltd., South China University of Technology, Guangzhou, China

**Keywords:** campus common spaces, healthy community, healthy environment design, college students, behavioral patterns, psychologically restorative effects

## Abstract

Contemporary college students are suffering from increasingly serious psychological health problems, such as attention fatigue, psychological stress and negative emotions. A growing body of evidence has revealed that restorative environment design is conducive to psychological health. As the main choice of venue for students’ daily activities, campus common spaces are supposed to be restorative to some extent. Given the above, the author studied 22 common spaces in the South China University of Technology (SCUT) Wushan Campus from the perspective of college students’ behavioral patterns based on theories pertaining to restorative environments, then constructed a structural equation model (SEM) analyzing the psychologically restorative effects exerted by the characteristics of campus common spaces upon college students through a scale design and questionnaire survey. With the analysis of 478 valid questionnaires, the research found that the characteristics of campus common spaces with psychologically restorative effects mainly comprise the architectural environment, landscape environment, rest facilities and activity facilities. Among them, the characteristics of activity facilities and the landscape environment have the greatest impact on psychologically restorative effects, accounting for 33 and 30% of the total effects, respectively; they are followed by those of the architectural environment, which accounts for 21% of the total effects; those of the rest facilities have the least impact, accounting for 16% of the total effects. The research also found that the characteristics of campus common spaces can both directly influence college students’ psychological recovery and produce psychologically restorative effects mediated by college students’ behavioral patterns. The mediation effect of college students’ behavioral patterns accounts for approximately 41% of the total effect of psychological restoration, in which the psychologically restorative effect of dynamic exercise behaviors is 2.5 times that of static leisure behaviors. The research reveals how the characteristics of campus common spaces promote the psychological restoration of college students, and it provides inspiration for healthy environment design in campus common spaces.

## 1. Introduction

College students are at a high risk of developing psychological conditions, according to relevant research. Global cross-sectional studies conducted from 2016 to 2020 indicated that 29–40% of college students experience psychological problems (anxiety, emotional disorders, academic stress, etc.), and the percentage is rising with each passing year (1–3). The *2022 National Depression Blue Book* reveals that 50% of the people with depressive disorder in China are students, with psychological stress as the main cause (4). Compared with other groups, college students are more likely to suffer from attention fatigue and psychological stress as they are required to spend a large amount of time acquiring professional knowledge and participating in scientific research projects, hence exerting a negative impact on psychological health (5). Relevant research has indicated that stress reduction and attention restoration are the key mechanisms that promote psychological health (6). Therefore, college students are in urgent need of means that can help to alleviate their attention fatigue, release their stress and regain their psychological health.

Campus common spaces, as a choice of location for daily activities, are crucial to the healthy development of college students, both in mind and body. However, the design of common spaces in many colleges still lags behind the psychological needs of students. Due to limited construction time, the development of college campuses in China tends to merely focus on the completion of main buildings and lacks the in-depth consideration of common spaces in terms of functional planning, activity planning and atmosphere fostering. This prevents the formation of an emotional bond between college students and the campus environment, leading to a low degree of space activity and involvement. The State Council, the National Health and Family Planning Commission and the Publicity Department of the CPC Central Committee have successively issued policies addressing the psychological needs of college students and the existing problems of campus spaces. Documents such as the *Outline of the Healthy China 2030 Plan and the Healthy China Action (2019–2030)* emphasize the task of building healthy campuses and provide guidelines for the psychological health of college students (7). It is evident that, faced with global crises (8, 9), psychological health is receiving increasingly more attention from the Chinese government. Building campus spaces that can facilitate college students’ psychological restoration has become an urgent need.

Scholars at home and abroad have, in recent years, participated in extensive discussions and studies on how to build a spatial environment conducive to psychological health. Relevant researchers have revealed that a restorative environment can effectively help individuals to restore their consumed attention, relieve psychological stress and embrace a series of positive changes in both body and mind (10, 11). There have already been research works confirming that campus common spaces can effectively accelerate the physical and psychological restoration of college students. Nonetheless, existing research mainly focuses on the evaluation and comparison of the restorative ability and restorative effects of different types of spaces, lacking systematic research on the restorative elements of campus common spaces. Current empirical studies mostly adopt simple linear relationships to explain the association between the campus environment and restorative effects. Their evidence is relatively simple, and they ignore the impact of students’ space use and behavioral patterns on psychologically restorative effects. Therefore, it is in some ways necessary for empirical studies to identify how the characteristics of campus common spaces affect college students’ behavioral patterns and consequently psychological restorative effects.

In this context, the author studied the South China University of Technology (SCUT) Wushan Campus and employed structural equation modeling (SEM) to analyze the results of college students’ psychological restoration contributed by the characteristics of campus common spaces under different behavioral patterns. The research attempts to answer the following questions: (1) Which characteristics of campus common spaces are conducive to college students’ psychological restoration? (2) Are the characteristics of campus common spaces and the mechanism promoting college students’ psychological restoration subject to the impact of college students’ behavioral patterns? What are the influence effects? (3) Based on the answers to the above two questions, are there suggestions for the design optimization of existing campus common spaces?

## 2. Literature review and research hypotheses

### 2.1. The theory of the restorative environment

**Figure 1:**
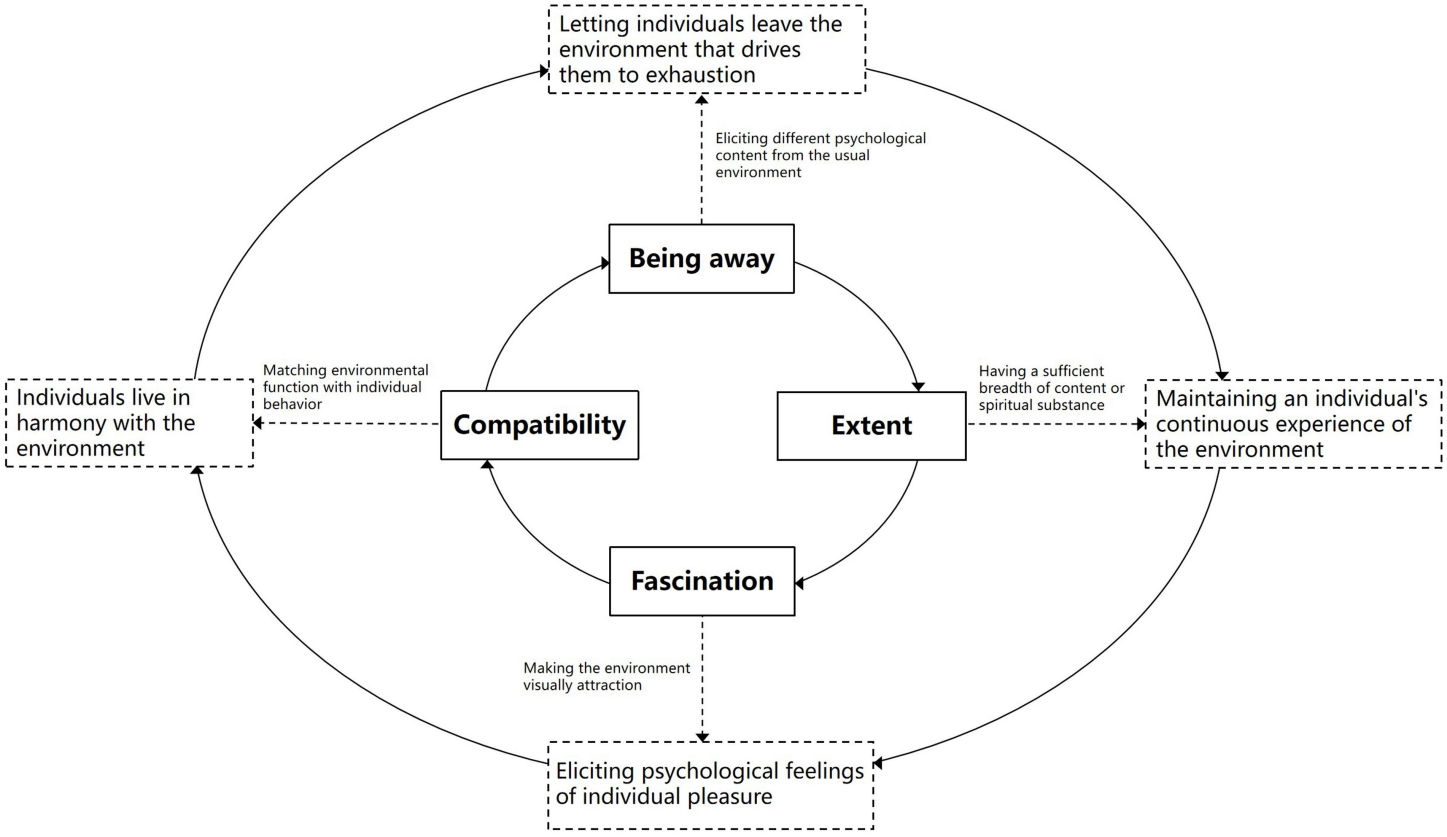
Four characteristics of restorative environment (15, 16).

“Restorative environment” refers to an environment that enables people to better recover from psychological fatigue and stress (12). Two major theories have been developed since this concept was proposed, i.e., Attention Restoration Theory (ART) and Stress Reduction Theory (SRT). The ART, proposed in 1989 by the Kaplans, who are renowned in the field of environmental psychology, pointed out that a decline in an individual’s ability to concentrate can lower their work or study efficiency and accuracy and lead to psychological fatigue; if the environment in which an individual exists is engaging in some way, it can help the individual to avoid fatigue-causing thought tasks and restore the consumed attention to a certain extent (13). The SRT, proposed by Ulrich, a scholar in the field of rehabilitation architecture, put forward that a clear state of stress perception can lead to negative emotions among individuals, as well as a decline in their cognitive and behavioral abilities (14). An environment that contains positive factors can effectively relax the individuals within it, reduce their sense of stress and transform negative emotions into positive ones. Kaplans et al. have proven that a restorative environment normally has four characteristics (Figure 1), i.e., being away, extent, fascination and compatibility (13), which contribute to individual attention restoration and stress reduction in a time-sequence-based progressive manner (15, 16).

### 2.2. Psychologically restorative effects of campus common spaces

Research by Kaplan shows that people constantly consume certain physiological, psychological and social resources in daily life, study and work, which produces a need for restoration when they are physically and psychologically exhausted (12, 13). Laumann and Bratman et al. pointed out that a spatial environment restores the psychological health of individuals mainly from two perspectives, i.e., “attention restoration,” specifically the improvement of attention and memory (17), and “stress reduction,” specifically the reduction of negative emotions and the promotion of positive emotions (18). According to research by Zhang, the resilience of a spatial environment refers to the ability of the characteristics and elements of a spatial environment to support the effective restoration of individuals, which determines whether individuals can obtain effective restoration in such a spatial environment (15). In campus common spaces, the characteristics of common spaces and the existence of their various elements will either facilitate or hinder the restoration of college students in these spaces, thus affecting their psychological restoration. Therefore, “the facilitating (or hindering) effect of the environmental characteristics of campus common space on the psychological restoration of college students” can be defined as “psychologically restorative effects.” The psychological restorative effects of campus common spaces are related to the characteristics of the spatial environment and the behavioral patterns of college students in using these spaces.

### 2.3. Characteristics of campus common spaces

Campus common spaces are where teachers and students live and communicate, referring to mainly campus landscape spaces (vegetation and waterfront landscape), squares, courtyards and sports fields. In the planning and design of college campuses, common spaces are spatial nodes of different scales and forms enclosed by campus buildings. Their quality can be upgraded through landscape design, and they are equipped with rest and activity facilities. Therefore, the characteristics of campus common spaces are mainly formed by those of the architectural environment, landscape environment and facility support.

In recent years, some scholars have begun to turn their attention to the restorative effect of the characteristics of the architectural environment. Japanese architect Ashihara studied the building enclosure width (D) and building height (H) in common spaces and concluded that the ratio of D/H = l ~ 2 is appropriate to elevate people’s positive emotions and spatial experiences (19). Based on machine learning simulation, Xiang et al. concluded that the shape and layout of building enclosures in common spaces are significantly related to people’s emotions (20). Lindal, Weber and Masullo et al. believed that the number of turns in architectural outlines and historical elements in the architectural environment, and rich changes in building facades, can arouse people’s interest and divert their attention from their daily needs and spiritual content and help them to achieve psychological restoration (21–26). In addition, there is ample evidence that increases in the quantity, type and color of vegetation in campus landscape spaces and sports fields is significantly related to enhanced psychological restoration (27–29). The degree of tree cover and green window views in campus have a significant positive relationship with the health, well-being and academic performance of college students (30, 31). For instance, Yang et al. believe that the type and distribution range of trees in a campus are related to the mental health levels of college students (32). Elsadek and Guo et al. proved through research that green and yellow plants can cause individuals to feel comfortable and peaceful, relieve stress and attention fatigue and improve work efficiency (33, 34). Wang et al. believed that extensive lawn spaces have restorative characteristics, and that looking at a green lawn free of people can evidently reduce stress and psychological fatigue (35). Research by Lu and Rout et al. shows that the accessibility and aesthetics of water bodies are significantly and positively associated with psychologically restorative effects (36, 37). Other researchers show that the rest facilities and activity facilities in campus common spaces can all significantly promote the psychological restoration of college students. For instance, research by Du and Nordh et al. shows that a sufficient number of comfortable and hygienic rest seats and seats with a natural view in campus common spaces can significantly increase the stay time and frequency of college students, which is conducive to producing restorative effects (38, 39). Research by Skärbäck shows that the number of activity facilities, and the compatibility and support of activity fields, can promote college students’ physical activities, helping them to relieve their psychological stress and restore their attention (40).

The above researchers prove that the characteristics of the architectural environment, landscape environment, rest facilities and activity facilities in campus common spaces can all significantly promote the psychological restoration of college students. Therefore, the author puts forward the following hypothesis: H1—The characteristics of campus common spaces have a direct and significant positive impact on the psychologically restorative effects upon college students.

### 2.4. College students’ behavioral patterns in campus common spaces

The environmental characteristics of campus common spaces have a significant impact on college students’ behavioral patterns. Hipp, Markevych and Wang et al. proposed that the quality of the landscape environment in campus common spaces is positively related to college students’ behavioral activities, and that a campus waterscape with a natural embankment is attractive to college students who prefer static rest activities such as reading, meditation and view appreciation, while a landscape with a hard pavement appeals more to those who enjoy dynamic exercise activities such as walking and running (41–43). Yu et al. put forward that sufficient sports fields and complete sports facilities on campus can secure the environment required for and promote college students’ dynamic exercise behaviors (44). In addition, research by Altaher et al. shows that highly comfortable rest facilities with views can facilitate college students’ static leisure behaviors such as meditation, reading and viewing, and help them to achieve emotional regulation and psychological restoration (45). As such, the author puts forward the following hypothesis: H2—The characteristics of campus common spaces have a direct and significant positive impact on college students’ behavioral patterns.

Relevant studies have shown that behavioral activities have higher health promotion effects. Research by Holt et al. shows that college students who actively participate in outdoor activities on campus on a regular basis tend to be energetic and less sensitive to stress (46). Pasanen, Yuan and Herranz-Pascual et al. proposed that memory and emotional restoration are positively correlated with physical activities, and they pointed out after their research that a 30-min walk or meditation on a square can remarkably improve emotion and attention, and that a walk in a natural environment can relieve stress and anxiety and improve cognitive levels (47–51). Chawla, Sun and Yang et al. found, through observation and interviews, that the leisure and exercises activities of college students in campus landscape spaces can positively affect their emotions, and they indicated that stress reduction and attention restoration are associated with the leisure activity choices of college students (52–54). Although some studies have shown that individuals who engage in dynamic behavior activities (such as fitness, ball games and running) in the same environment enjoy greater restoration benefits than those performing static behavior activities (such as meditation) (55), the ART proposed by Kaplan demonstrates that behavioral patterns either supported by the environment or with a high degree of feedback can add to the restoration benefits (56). Static leisure behaviors (such as meditation, breathing in fresh air, contact with nature, etc.) can improve the level of restoration from psychological stress by promoting the collaboration between multiple senses (vision, hearing, and smell) (57). Based on the above research, the author puts forward the following hypothesis: H3—Behavioral patterns (static and dynamic exercise behaviors) have a direct and significant positive impact on the psychological restoration effects upon college students.

According to existing studies, the characteristics of the architectural environment, landscape environment, rest facilities and activity facilities in campus common spaces can promote the psychological restoration of college students. The characteristics of campus common spaces give rise to different behavioral patterns among college students, which lead to varied restorative effects. It can be inferred that college students’ behavioral patterns play a mediating role in the relationship between the characteristics of campus common spaces and the psychologically restorative effects, hence leading to the following hypothesis: H4—The characteristics of campus common spaces can produce a positive impact on psychologically restorative effects through the mediation of college students’ behavioral patterns.

**Figure 2:**
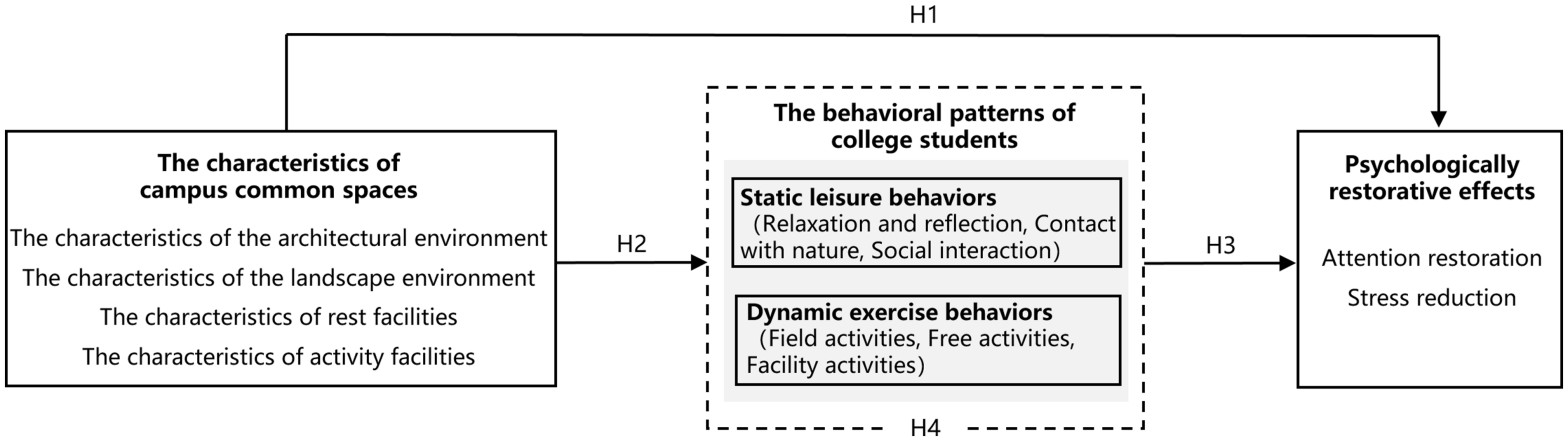
Hypothetical model of the psychologically restorative effects of campus common spaces.

The hypothesis model (Figure 2) of this research was produced based on the above hypotheses.

Based on the hypothesis model, the aforesaid four research hypotheses are divided into four sets of hypotheses (Table 1).

**Table 1:** Research hypotheses of the psychologically restorative effects of campus common spaces.

No.	Research hypotheses
H1	The characteristics of campus common spaces have a direct and significant positive impact on the psychologically restorative effects upon college students.
H1a	The characteristics of the architectural environment have a direct and significant positive impact on the psychologically restorative effects upon college students.
H1b	The characteristics of the landscape environment have a direct and significant positive impact on the psychologically restorative effects upon college students.
H1c	The characteristics of rest facilities have a direct and significant positive impact on the psychologically restorative effects upon college students.
H1d	The characteristics of activity facilities have a direct and significant positive impact on the psychologically restorative effects upon college students.
H2	The characteristics of campus common spaces have a direct and significant positive impact on the behavioral patterns of college students.
H2a1	The characteristics of the architectural environment have a direct and significant positive impact on the static leisure behaviors of college students.
H2a2	The characteristics of the landscape environment have a direct and significant positive impact on the static leisure behaviors of college students.
H2a3	The characteristics of rest facilities have a direct and significant positive impact on the static leisure behaviors of college students.
H2b1	The characteristics of the architectural environment have a direct and significant positive impact on the dynamic exercise behaviors of college students.
H2b2	The characteristics of the landscape environment have a direct and significant positive impact on the dynamic exercise behaviors of college students.
H2b3	The characteristics of activity facilities have a direct and significant positive impact on the dynamic exercise behaviors of college students.
H3	College students’ behavioral patterns have a direct and significant positive impact on the psychologically restorative effects upon college students.
H3a	Static leisure behaviors have a direct and significant positive impact on the psychologically restorative effects upon college students.
H3b	Dynamic exercise behaviors have a direct and significant positive impact on the psychologically restorative effects upon college students.
H4	The characteristics of campus common spaces can produce a positive impact on the psychologically restorative effects upon college students through the mediation of their behavioral patterns.
H4a1	The characteristics of the architectural environment can produce a positive impact on the psychologically restorative effects upon college students through the mediation of their static leisure behaviors.
H4a2	The characteristics of the architectural environment can produce a positive impact on the psychologically restorative effects upon college students through the mediation of their dynamic exercise behaviors.
H4b1	The characteristics of the landscape environment can produce a positive impact on the psychologically restorative effects upon college students through the mediation of their static leisure behaviors.
H4b2	The characteristics of the landscape environment can produce a positive impact on the psychologically restorative effects upon college students through the mediation of their dynamic exercise behaviors.
H4c	The characteristics of rest facilities can produce a positive impact on the psychologically restorative effects upon college students through the mediation of their static leisure behaviors.
H4d	The characteristics of activity facilities can produce a positive impact on the psychologically restorative effects upon college students through the mediation of their dynamic exercise behaviors.

## 3. Research methodology

### 3.1. Overview of the researched area

**Figure 3:**
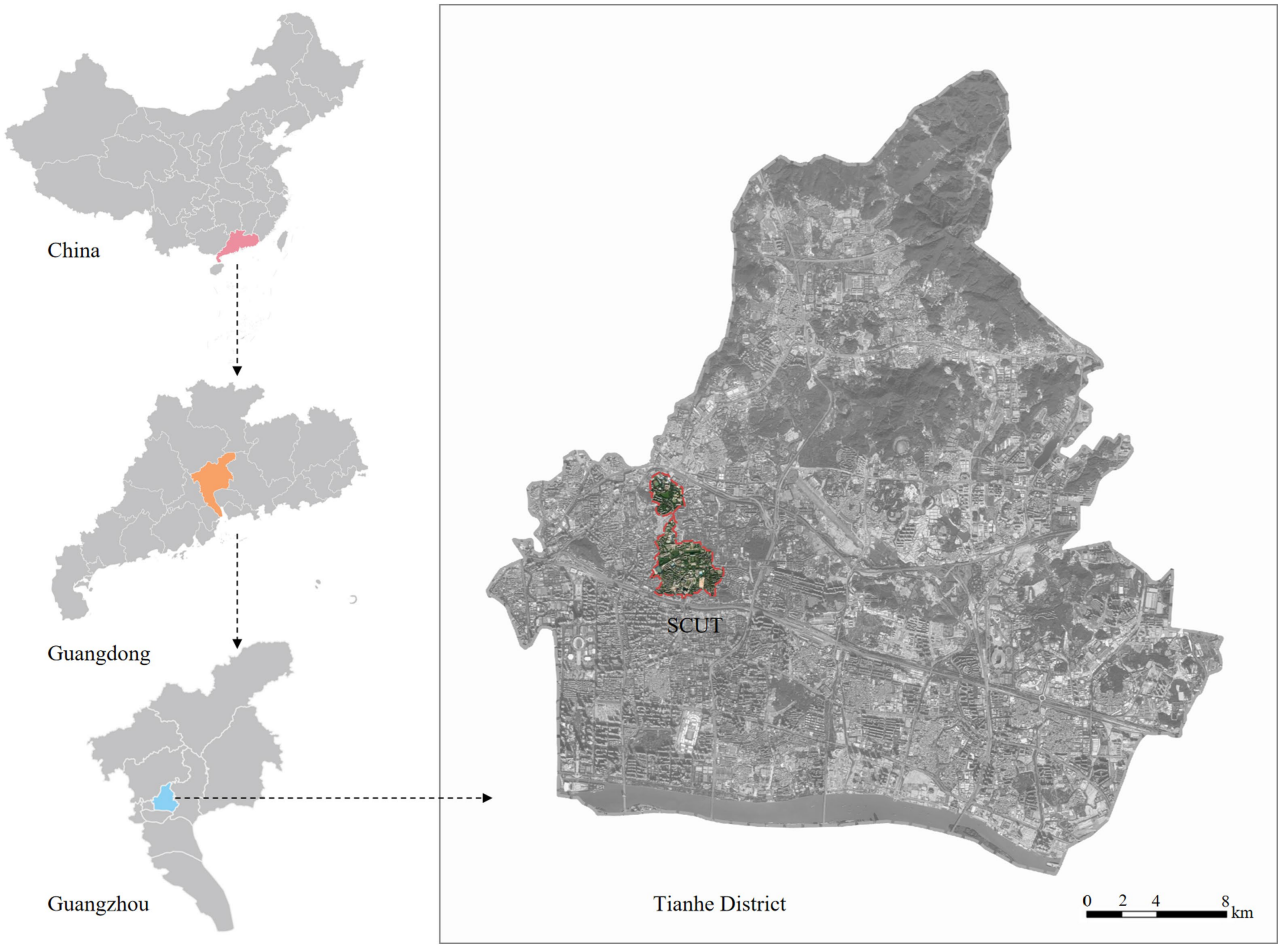
Location map of SCUT Wushan campus.

**Figure 4:**
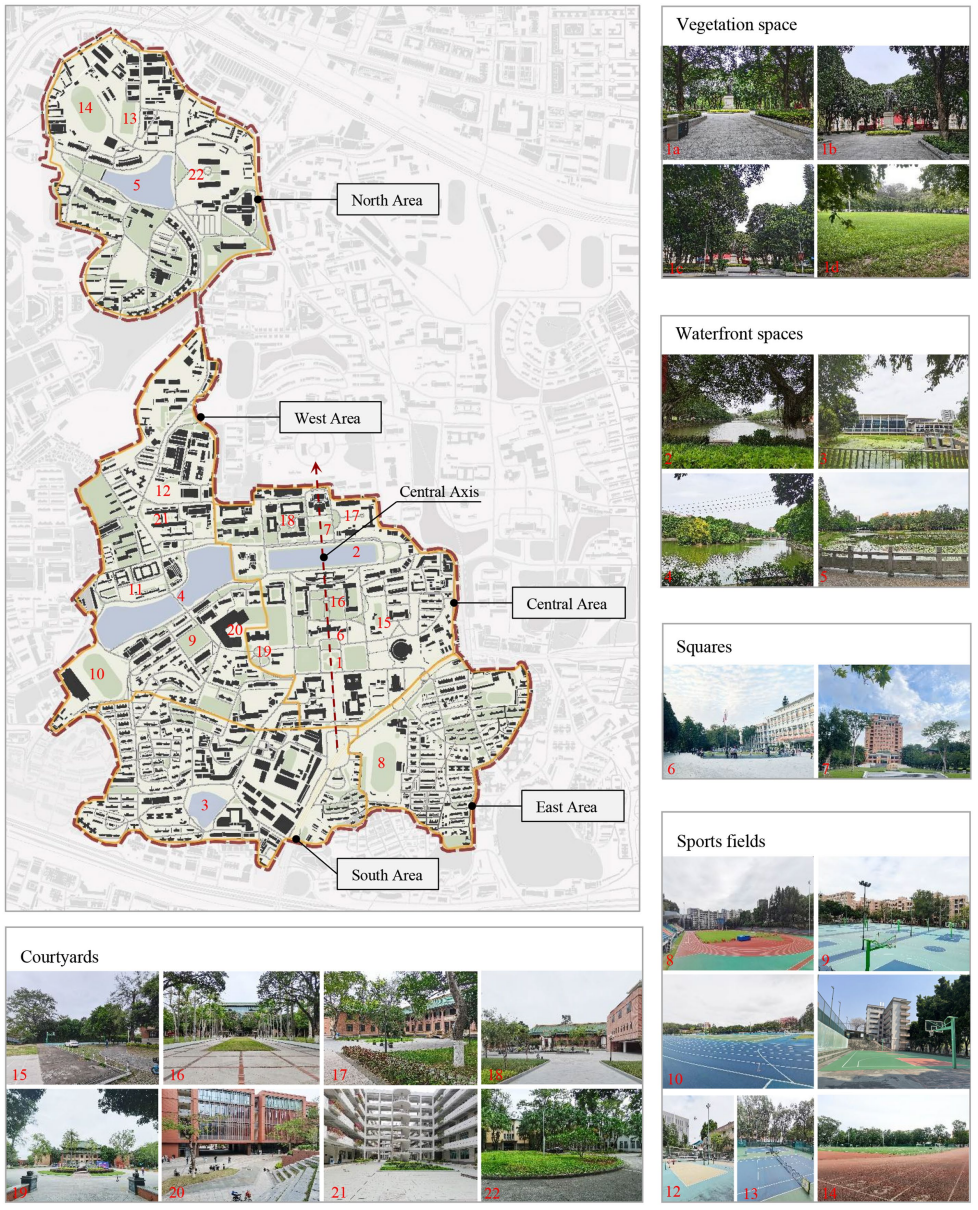
Master plan of SCUT Wushan campus and 22 researched common spaces.

The SCUT Wushan Campus is located in Tianhe District (Figure 3), Guangzhou, accommodating approximately 29,000 students on its 1.83 million square meters of land. The campus features an integrated north–south central axis extending from the south gate to the Liwu SciTech Building. It is divided into five areas: the Central Area, East Area, South Area, West Area and North Area. The campus common spaces are composed of vegetation landscape spaces, waterfront landscape spaces, squares, courtyards and sports fields (Figure 4). The research covered 9 sites in the Central Area, 7 in the West Area, 4 in the North Area, 1 in the South Area and 1 in the East Area, all selected based on the distribution characteristics of campus functions and college students’ extracurricular activities. These 22 common spaces included 1 vegetation space, 4 waterfront spaces, 2 squares, 8 courtyards and 7 sports fields. The research targeted the characteristics of the architectural environment, landscape environment, rest facilities and activity facilities in these spaces, as well as the behavioral activities of and the psychologically restorative effects upon the college students in these spaces. The characteristics of the common spaces selected in the research are typical, covering all types of campus common spaces, hence providing a representative research sample.

### 3.2. Questionnaire design

The measurement scale in this research is based on the results of previous research performed by others, with some measurement items adjusted and redeveloped as per the hypothesis model (58). The quantity of measurement items references the widely recognized standards in the academic community, i.e., 2 indicators are acceptable, 3 indicators are better, and 4 indicators are the best (59). Therefore, in the scale developed for the research, each measurement variable contains 3–4 measurement indicators, which constitute a total of 7 measurement indicators. The Likert 5-level scale evaluation method was adopted, where “1” means strongly disagree, “2” means disagree, “3” means neutral, “4” means agree and “5” means strongly agree. The surveyed college students rated relevant descriptions based on their true ideas. In January 2022, the author distributed the questionnaires in the common spaces of the SCUT Wushan Campus and retrieved a total of 133 valid ones for pre-research; then, based on the results of the pre-research, the author amended the measurement scale and eventually determined the formal research scale and questionnaire.

### 3.3. Data collection and analysis methodology

In October 2022, the author conducted a formal questionnaire survey at the SCUT Wushan Campus, randomly distributing paper questionnaires to college students at the 22 surveyed sites. According to the principle wherein the ratio of the sample size to the quantity of observed variables should be at least 10:1 ~ 15:1 (60), a total of 500 questionnaires were distributed, while 478 valid ones were retrieved, hence yielding an effective retrieval rate of 95.6%. The specific composition of the retrieved questionnaire samples shows that the surveyed students were reasonably distributed in terms of age, grade and major and relatively evenly distributed in gender, so the overall sample structure was reasonable and fairly representative.

**Figure 5:**
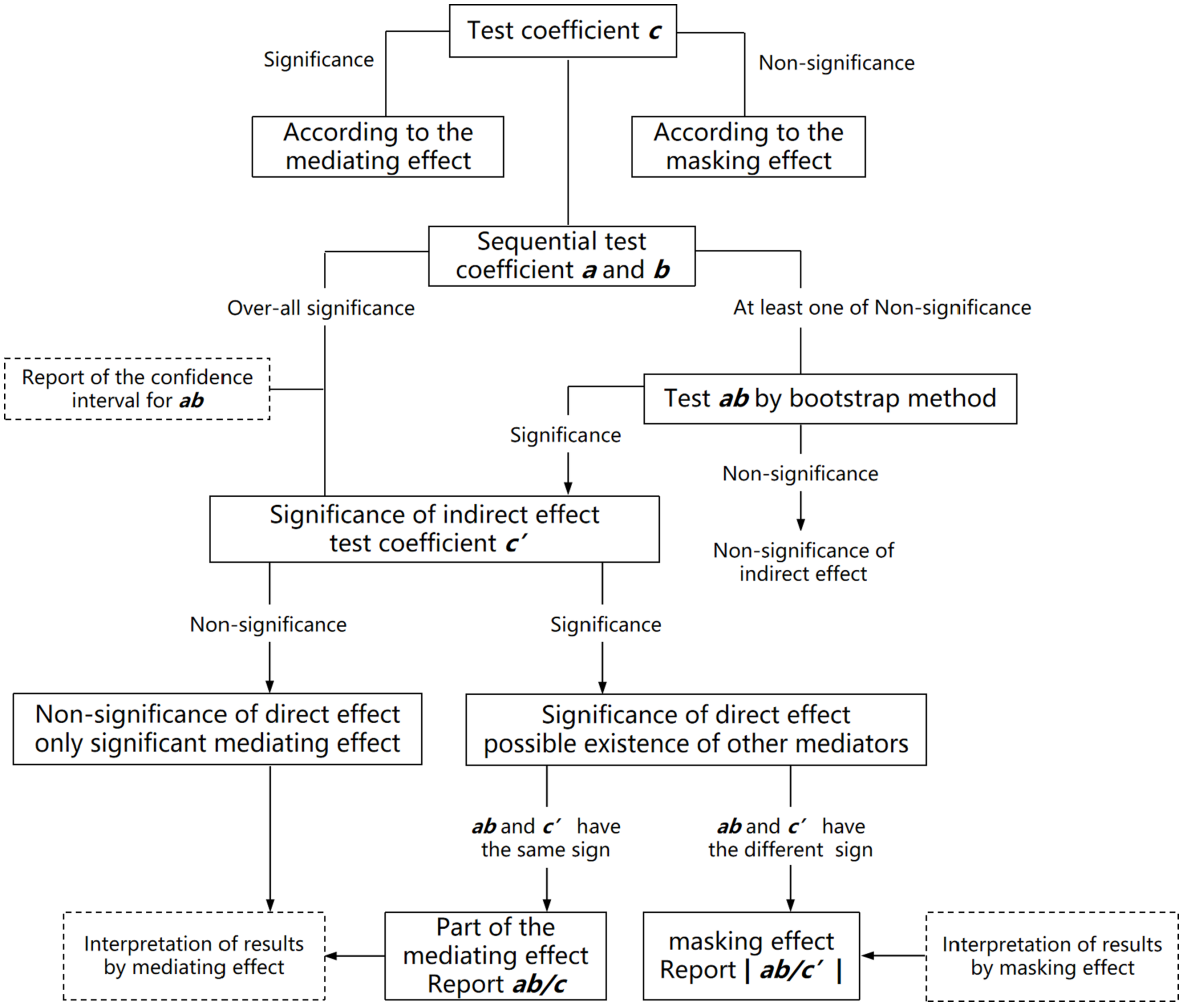
Test process of mediation effects (61). Coefficient *c*: the total effect of an independent variable on a dependent variable; Coefficient *a*: the effect of an independent variable on a mediating variable; Coefficient *b*: the effect of a mediating variable on a dependent variable after the influence of the independent variable is controlled; Coefficient *c*’: the direct effect of an independent variable on a dependent variable after the influence of the mediating variable is controlled.

Structural equation modeling (SEM) was adopted to test the previously proposed hypothesis model. First, the influencing factors and path coefficients of the restorative effects of campus common spaces were analyzed quantitatively; second, the mediation effects of college students’ behavioral patterns were tested. The most popular approach to mediation effect testing is the Baron and Kenny method, which, however, has been criticized and questioned continuously in recent years. Therefore, the more widely recognized Bootstrap method was employed instead for a direct test of the coefficient product. Chinese scholars Wen et al. have analyzed relevant topics and summarized the specific mediation effect analysis process (61) (Figure 5), which was referred to in this research.

## 4. Model test and results analysis

### 4.1. Analysis of data reliability and validity

The survey data were brought into the SPSS 26.0 and AMOS 26.0 statistical software for confirmatory factor analysis (CFA), which tests data reliability and validity. According to the test results, the Cronbach’s *α* coefficient of the total scale is 0.958, while those of all latent variables are above 0.85, indicating that the observed variables for each latent variable are well designed, hence indicating the relatively high reliability of the questionnaire. The Bartlett’s sphericity test and KMO value analysis of the survey data showed that the *p*-value was 0.000 (*p* < 0.001), passing Bartlett’s sphericity test, and the KMO value was 0.949, greater than 0.7, meaning that the sample data were suitable for factor analysis. As shown in Table 2, the factor loads of all observed variables on the corresponding latent variables were greater than the standard value of 0.5, indicating a statistically significant subordination between the latent variables and the observed variables. In the overall correlation analysis of the project, the corrected item-total correlation (CITC) coefficients were all greater than 0.4, and the composite reliability (CR) coefficients of all latent variables were greater than 0.7, denoting the relatively high internal consistency of the measurement questions of each latent variable. The average of variance extracted (AVE) values of all latent variables were greater than 0.5, indicating the relatively high convergence validity of the measurement variables.

**Table 2:** Analysis results of model reliability, validity, and CFA.

Latent variables	Observed variables	Standardized factor loadings	CITC	C.R.	AVE	Cronbach’s *α*
F1 The characteristics of the architectural environment	A1 Appropriate scale of building enclosure	0.806	0.625	0.893	0.676	0.891
	A2 Diverse forms of building enclosure	0.850	0.584			
	A3 Strong architectural historical atmosphere	0.805	0.637			
	A4 Varied building facades	0.661	0.606			
F2 The characteristics of the landscape environment	A5 Abundant plant types	0.806	0.674	0.916	0.733	0.915
	A6 Rich plant colors	0.778	0.688			
	A7 Extensive lawn coverage	0.797	0.634			
	A8 Highly ornamental waterscape	0.773	0.659			
F3 The characteristics of rest facilities	A9 Plentiful rest facilities	0.791	0.615	0.903	0.700	0.901
	A10 Comfortable rest facilities	0.848	0.627			
	A11 Rest facilities with view	0.809	0.597			
	A12 Hygienic rest facilities	0.748	0.603			
F4 The characteristics of activity facilities	A13 Plentiful activity fields	0.820	0.701	0.906	0.706	0.902
	A14 Abundant types of activity fields	0.780	0.674			
	A15 Accessible activity fields	0.794	0.673			
	A16 Sufficient number of fitness facilities	0.675	0.664			
F5 Static leisure behaviors	B1 Relaxation and reflection	0.793	0.625	0.890	0.729	0.890
	B2 Contact with nature	0.761	0.660			
	B3 Social interaction	0.813	0.601			
F6 Dynamic exercise behaviors	B4 Field activities	0.566	0.757	0.887	0.723	0.887
	B5 Free activities	0.686	0.729			
	B6 Facility activities	0.700	0.716			
F7 Psychologically restorative effects	C1 Restoration of consumed energy	0.760	0.737	0.915	0.730	0.922
	C2 Mitigation of psychological fatigue	0.687	0.805			
	C3 Relief of anxiety and stress	0.678	0.741			
	C4 Regulation of negative emotions	0.689	0.760			

*N* = 478.

### 4.2. Model test

**Figure 6:**
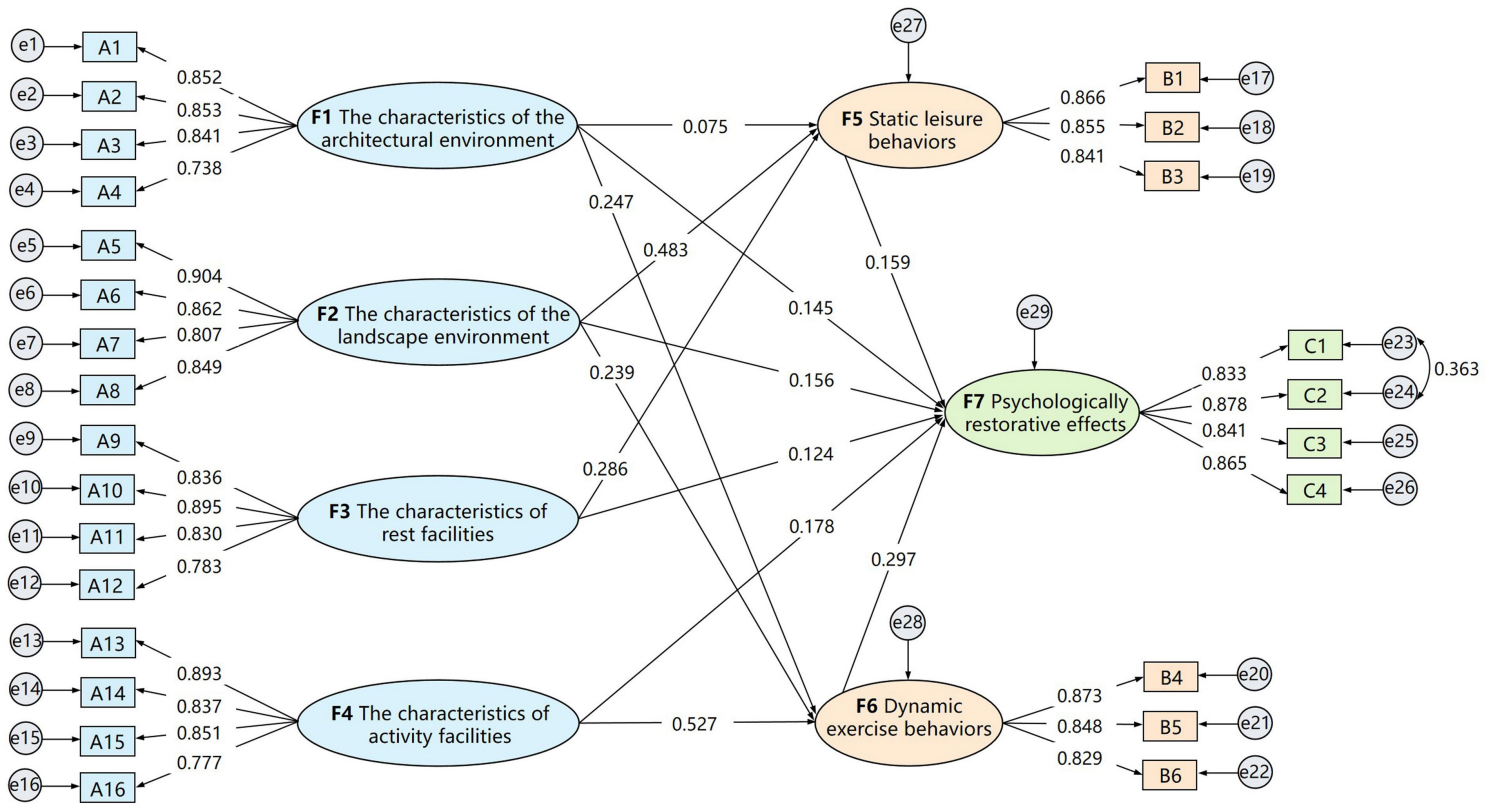
Diagram of standardized parameter estimation paths of the model for the psychologically restorative effects of campus common spaces.

In this research, the parameter estimation result and standardized path coefficients (Figure 6) of the model were obtained using the maximum likelihood estimation (MLE) method. From the fit indices of the measurement model (Appendix 1), it can be seen that the χ2/df value was 1.993, smaller than 2, meaning that the model was well fitted; the GFI and AGFI values were 0.921 and 0.901, respectively, both greater than the recommended value of 0.9, indicating that the model was acceptable; the RMSEA value was smaller than 0.05, and the CFI, NFI and IFI values were all greater than 0.9, so the goodness of fit was relatively high. The above values proved that the structural equation model constructed was relatively ideal, and the hypothesis model had a fairly good fit measure overall.

### 4.3. Analysis of path coefficients

The magnitude of the standardized path coefficients shows the relationship between the measurement variables and the influence degree of each measurement indicator. Whether the path coefficients between the measurement variables are significant can be simply judged by the *t*-value test and *p*-value. Specifically, as long as the *t*-value is greater than 1.96 or the *p*-value is smaller than 0.05, the path coefficients can be deemed significant. It can be seen from the test results of the path coefficients (Table 3) that hypothesis H2a1 (the characteristics of the architectural environment have a significant positive impact on static leisure behaviors) is not accepted, while the other hypothesis results are valid.

**Table 3:** Analysis results of paths between variables.

Hypothesis	Regression path of the measurement model	Unstandardized estimate	Standardized estimate	S.E.	C.R. (*t*-value)	*p*	Conclusion
H1a	F1 → F7	0.136	0.145	0.043	3.140	0.002	Supported
H1b	F2 → F7	0.152	0.156	0.050	3.046	0.002	Supported
H1c	F3 → F7	0.134	0.124	0.047	2.879	0.004	Supported
H1d	F4 → F7	0.168	0.178	0.057	2.940	0.003	Supported
H2a1	F1 → F5	0.075	0.075	0.046	1.628	0.104	Not supported
H2a2	F2 → F5	0.499	0.483	0.055	9.151	***	Supported
H2a3	F3 → F5	0.330	0.286	0.059	5.599	***	Supported
H2b1	F1 → F6	0.251	0.247	0.045	5.566	***	Supported
H2b2	F2 → F6	0.249	0.239	0.041	6.107	***	Supported
H2b3	F4 → F6	0.534	0.527	0.049	10.975	***	Supported
H3a	F5 → F7	0.149	0.159	0.045	3.289	0.001	Supported
H3b	F6 → F7	0.276	0.297	0.068	4.023	***	Supported

“***” Means significant at level 0.001; *N* = 478.

Table 3 shows that the standardized path coefficients of F1 (the characteristics of the architectural environment), F2 (the characteristics of the landscape environment) and F3 (the characteristics of rest facilities) to F5 (static leisure behaviors) are 0.75, 0.483, and 0.286, respectively. The *p*-value of F1 (the characteristics of the architectural environment) is 0.104, greater than 0.05, which is insignificant, indicating that F1 (the characteristics of the architectural environment) has no significant impact on F5 (static leisure behaviors). The *p*-values of F2 (the characteristics of the landscape environment) and F3 (the characteristics of rest facilities) are both smaller than 0.05, reaching a significant level, indicating that F2 (the characteristics of the landscape environment) and F3 (the characteristics of rest facilities) both have a significant positive impact on F5 (static leisure behaviors). Among them, F2 (the characteristics of the landscape environment) has the largest standardized path coefficient, meaning that F2 (the characteristics of the landscape environment) has the greatest impact on F5 (static leisure behaviors). The standardized path coefficients of F1 (the characteristics of the architectural environment), F2 (the characteristics of the landscape environment) and F4 (the characteristics of activity facilities) to F6 (dynamic exercise behaviors) are 0.247, 0.239, and 0.527, respectively. Their *p*-values are all smaller than 0.05, reaching a significant level, indicating that F1 (the characteristics of the architectural environment), F2 (the characteristics of the landscape environment) and F4 (the characteristics of activity facilities) all have a significant positive impact on F6 (dynamic exercise behaviors). Among them, F4 (the characteristics of activity facilities) has the largest standardized path coefficient, meaning that F4 (the characteristics of activity facilities) has the greatest impact on F6 (dynamic exercise behaviors).

The standardized path coefficients of F1 (the characteristics of the architectural environment), F2 (the characteristics of the landscape environment), F3 (the characteristics of rest facilities) and F4 (the characteristics of activity facilities) to F7 (psychological restorative effects) are 0.145, 0.156, 0.124, and 0.178, respectively. Their *p*-values are all smaller than 0.05, reaching a significant level, indicating that F1 (the characteristics of the architectural environment), F2 (the characteristics of the landscape environment), F3 (the characteristics of rest facilities) and F4 (the characteristics of activity facilities) all have a direct impact on F7 (psychologically restorative effects). Among them, F4 (the characteristics of activity facilities) has the largest standardized path coefficient, meaning that F4 (the characteristics of activity facilities) has the greatest direct impact on F7 (psychologically restorative effects).

The standardized path coefficients of F5 (static leisure behaviors) and F6 (dynamic exercise behaviors) to F7 (psychologically restorative effects) are 0.159 and 0.297, respectively. Their *p*-values are both smaller than 0.05, reaching a significant level, indicating that F5 (static leisure behaviors) and F6 (dynamic exercise behaviors) both have a significant positive impact on F7 (psychologically restorative effects). Among them, F6 (dynamic exercise behaviors) has the largest standardized path coefficient, meaning that F6 (dynamic exercise behaviors) has the greatest impact on F7 (psychologically restorative effects). Based on the analysis of the above path coefficients, the structural equation model diagram supported by the data was drawn, as shown in Appendix 2.

### 4.4. Test of mediation effects

In this study, the mediation effect analysis process summarized by Wen et al. was adopted to test the mediation effects of college students’ behavioral patterns. The Bootstrap method was employed to acquire 5,000 samples, obtain the standardized estimated values and standard errors among the variables and eventually calculate the significance level of the total effects, direct effects and indirect effects (Table 4). As long as the bias-corrected percentile and percentile estimated effect sizes do not contain 0 within the lower limit and upper limit of the 95% confidence interval, the *z*-value is no smaller than 1.96 and the Sig (two-tailed) *p*-value is smaller than 0.05, the effect size can be deemed significant. First, according to the results of the total effects in Table 4, independent variables F1 (the characteristics of the architectural environment), F2 (the characteristics of the landscape environment), F3 (the characteristics of rest facilities) and F4 (the characteristics of activity facilities), respectively, have a significant total effect on dependent variable F7 (psychologically restorative effects). Their lower and upper limit values do not contain 0, their *z*-values are all greater than 1.96 and the *p*-values are all significant at level 0.05. Second, according to the results of the direct effects in Table 4, only the lower and upper limit values of the standardized direct effect of F1 (the characteristics of the architectural environment) to F5 (static leisure behaviors) contain 0, the *z*-value of F1 is smaller than 1.96 and the *p*-value is greater than 0.05. The results are insignificant, consistent with the results of the abovementioned path analysis indicating that the F1 → F5 hypothesis is not valid, while the other hypotheses are valid. Finally, according to the test results of the indirect effects, the indirect effect *z*-values of independent variables F1 (the characteristics of the architectural environment), F2 (the characteristics of the landscape environment), F3 (the characteristics of rest facilities) and F4 (the characteristics of activity facilities), respectively, to dependent variable F7 (psychologically restorative effects) through mediating variables F5 (static leisure behaviors) and F6 (dynamic exercise behaviors) are 1.091, 1.973, 2.139, 2.219, 1.960, and 2.294, respectively, and their *p*-values are 0.138, 0.024, 0.016, 0.013, 0.025, and 0.011, respectively. Only the lower and upper limit values of the standardized indirect effect of F1 (the characteristics of the architectural environment) to F7 (psychologically restorative effects) through F5 (static leisure behaviors) contain 0, the *z*-value is smaller than 1.96 and the *p*-value is greater than 0.05. The results are insignificant; the path hypothesis of F1 → F5 → F7 is not valid, while other hypotheses are valid.

**Table 4:** Direct, indirect and total effects of the standardized hypothesis model.

Hypothesis	Path	Standardized estimate	Product of coefficient		Bootstrapping				*P* (Two-tailed significance)	Conclusion
					Bias-corrected Percentile 95%CI		Percentile 95%CI			
			SE	*Z*	Lower	Upper	Lower	Upper		
*Standardized direct effects*										
H2a1	F1 → F5	0.075	0.053	1.415	−0.029	0.180	−0.026	0.182	0.079	Not supported
H2b1	F1 → F6	0.247	0.059	4.186	0.131	0.364	0.135	0.371	0.000	Supported
H1a	F1 → F7	0.145	0.068	2.132	0.015	0.282	0.014	0.281	0.017	Supported
H2a2	F2 → F5	0.483	0.070	6.900	0.344	0.618	0.342	0.618	0.000	Supported
H2b2	F2 → F6	0.239	0.053	4.509	0.141	0.350	0.137	0.341	0.000	Supported
H1b	F2 → F7	0.156	0.069	2.261	0.021	0.292	0.020	0.291	0.012	Supported
H2a3	F3 → F5	0.286	0.074	3.865	0.141	0.432	0.133	0.427	0.000	Supported
H1c	F3 → F7	0.124	0.059	2.102	0.010	0.248	0.009	0.247	0.018	Supported
H2b3	F4 → F6	0.527	0.061	8.639	0.406	0.647	0.404	0.646	0.000	Supported
H1d	F4 → F7	0.178	0.083	2.145	0.023	0.352	0.005	0.332	0.016	Supported
H3a	F5 → F7	0.159	0.069	2.304	0.034	0.302	0.028	0.296	0.011	Supported
H3b	F6 → F7	0.297	0.119	2.496	0.077	0.543	0.084	0.554	0.006	Supported
*Standardized indirect effects*										
H4a1	F1 → F5 → F7	0.012	0.011	1.091	−0.002	0.043	−0.003	0.039	0.138	Not supported
H4a2	F1 → F6 → F7	0.073	0.037	1.973	0.018	0.161	0.017	0.160	0.024	Supported
H4b1	F2 → F5 → F7	0.077	0.036	2.139	0.018	0.160	0.013	0.151	0.016	Supported
H4b2	F2 → F6 → F7	0.071	0.032	2.219	0.021	0.149	0.018	0.142	0.013	Supported
H4c	F3 → F5 → F7	0.045	0.023	1.960	0.012	0.104	0.008	0.095	0.025	Supported
H4d	F4 → F6 → F7	0.156	0.068	2.294	0.043	0.310	0.042	0.310	0.011	Supported
*Standardized total effects*										
	F1 → F7	0.218	0.062	3.516	0.109	0.356	0.114	0.363	0.000	
	F2 → F7	0.304	0.061	4.984	0.191	0.432	0.190	0.430	0.000	
	F3 → F7	0.169	0.064	2.641	0.058	0.307	0.052	0.300	0.004	
	F4 → F7	0.334	0.070	4.771	0.202	0.473	0.191	0.465	0.000	

Standardized estimation of 5,000 bootstrap samples;“0.000” means significant at level 0.001; *N* = 478.

The above results show that the characteristics of campus common spaces, i.e., F1 (the characteristics of the architectural environment), F2 (the characteristics of the landscape environment), F3 (the characteristics of rest facilities) and F4 (the characteristics of activity facilities), respectively, have significant total and direct effects on F7 (psychologically restorative effects), and significant mediation effects through college students’ behavioral patterns F5 (static leisure behaviors) and F6 (dynamic exercise behaviors). This indicates that the mediation effects in this research are incomplete, and that the college students’ behavioral patterns play only a partial mediation role. As indicated in Table 4, the total effect exerted by the characteristics of campus common spaces on college students’ psychological restoration is 1.025, the direct effect is 0.603, and the mediation effect of college students’ behavioral patterns is 0.422, indicating that the characteristics of campus common spaces are improved by 1 unit, while the psychological restoration effects upon college students are improved by 1.025 units (with 0.422 being the effect exerted by the characteristics of campus common spaces on college students’ psychological restoration through their behavioral patterns and 0.603 being the direct effect exerted by the characteristics of campus common spaces on college students’ psychological restoration), so the mediation effect accounts for approximately 41% of the total effects. However, most of the existing research only focuses on the direct effect exerted by the characteristics of campus common spaces on college students’ psychological restoration, ignoring the mediation effects of college students’ behavioral patterns, which should be given more attention in future research. The mediation effect sizes of F5 (static leisure behaviors) and F6 (dynamic exercise behaviors) are 0.122 and 0.300, respectively. Among them, F6 (dynamic exercise behaviors) has the largest mediation effect size, meaning that F6 (dynamic exercise behaviors) has the greatest mediation effect. The psychologically restorative effect produced by the mediating path of dynamic exercise behaviors is 2.5 times that by the static leisure behavior path. Therefore, the dynamic exercise behavioral pattern should be considered in the design of campus common spaces.

## 5. Discussions

The research proposes four sets of hypotheses, and the empirical research results well support the hypothesis model. The research confirms that there are three paths for campus common spaces to influence the psychologically restorative effects upon college students, namely “Path 1: characteristics of campus common spaces → psychological restorative effects,” “Path 2: characteristics of campus common spaces → static leisure behaviors → psychological restorative effects,” and “Path 3: characteristics of campus common spaces → dynamic exercise behaviors → psychological restorative effects.” Based on the above three paths, the paper discusses the psychological restoration influence paths and the effects of the characteristics of the architectural environment, landscape environment, rest facilities and activity facilities in campus common spaces, and concludes that more restorative campus common spaces can be designed according to the characteristics of college students’ behavioral patterns.

### 5.1. Influence paths and effects of the characteristics of the architectural environment

**Figure 7:**
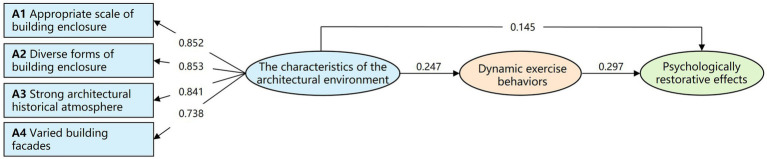
Influence effect illustration of the characteristics of the architectural environment.

The effect size of the characteristics of the architectural environment to psychological restorative effects is 0.218 (Table 4), accounting for 21% of the total effects. Through the comparison of the path coefficients in Figure 7, it can be seen that the effect size of the characteristics of the architectural environment to dynamic exercise behaviors is 0.247. Among the four measurement indicators, the path coefficients of “A2 Diverse forms of building enclosure” (0.853) and “A1 Appropriate scale of building enclosure” (0.852) in the characteristics of the architectural environment are the highest. This result indicates that the building enclosure form and scale are the factors that have the greatest impact on the dynamic exercise behaviors of college students. Previous researchers have not satisfactorily revealed the effects of students’ behavioral activities on psychological recovery. This research confirmed that an appropriate spatial scale can promote college students’ dynamic exercise behaviors, promoting psychological restoration through the mediation of dynamic exercise behaviors. The research also shows that historical buildings in campus common spaces can facilitate the free activities of college students, such as walking, playing and picture-taking, and have a positive impact on emotions and restoration from stress. This is consistent with the research results of Weber, Masullo, Reece and Guo, which revealed that architectural environments with a strong historical and cultural atmosphere are conducive to enhancing people’s spatial experiences and promoting psychological restoration (22–25).

In addition, the research results show that varied building facades have a relatively small impact on the dynamic exercise behaviors of college students. However, research on the urban streetscape by Lindal and Yang showed that the degree of variation and number of decorations on building facades are significantly related to attention restoration (21, 26). The main reason for this discrepancy is that the environment of campus spaces and that of the urban streetscape differ significantly in characteristics. Facade forms in the urban streetscape are diverse and dynamic; thus, they can notably attract visual attention and guide behavioral activities. In the planning and design of university campuses, architectural interfaces and building heights are subject to unified design standards, so facade forms tend to be unified and less changeable, having little impact on college students’ vision and behavioral activities.

### 5.2. Influence paths and effects of the characteristics of the landscape environment

**Figure 8:**
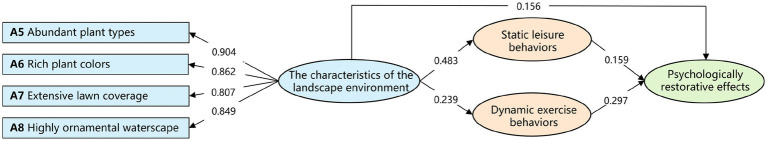
Influence effect illustration of the characteristics of the landscape environment.

The research results show that the total effect size of the characteristics of the landscape environment to psychological restorative effects is 0.304 (Table 4), accounting for 30% of the total effects. According to the comparison of the path coefficients in Figure 8, the effect sizes of the characteristics of the landscape environment to static leisure behaviors and dynamic exercise behaviors are 0.483 and 0.239, respectively, indicating that the influence effect of the characteristics of the landscape environment on college students’ static leisure behaviors is higher than that on dynamic exercise behaviors. Previous research mostly adopted simple linear relationships to explain the association between the characteristics of the campus landscape environment and restorative effects, which ignore the impact of students’ space use and behavioral patterns on the psychologically restorative effects. This research further demonstrates that the characteristics of the campus landscape environment and the mechanism promoting college students’ psychological restoration are subject to the impact of college students’ behavioral patterns by examining the mediating effect of college students’ behavioral activities. Among the path coefficients of the four measurement indicators of the characteristics of the landscape environment, “A5 Abundant plant types” (0.904) is the most important influencing factor, indicating that plant type has the greatest impact on the static leisure and dynamic exercise behaviors of college students. The research also shows that a visually pleasant waterscape can promote the static leisure and dynamic exercise behaviors of college students, who would frequently sit around the waterscape to relax, walk around, get together and chat. However, college students pay more attention to plant elements in common spaces, such as whether the proportion of green plants is high enough and whether the mix of plant types is rich. A survey by Lu and Fu showed that college students prefer campus water bodies to green plants (36). The reason for this difference could possibly be that the Wushan Campus is located in a climate zone with hot summers and warm winters, where college students tend to seek shaded spaces with abundant greenery when they are outdoors (62).

In addition, the research results indicate that the lawn coverage area has a relatively small impact on the static leisure and dynamic exercise behaviors of college students, which is consistent with the empirical results presented by Ha and Kim, i.e., plant and waterfront landscapes with high biodiversity on campus can better relieve stress and promote restoration than traditional lawns (63). The main reason is probably that lawns in many campus common spaces only serve aesthetic purposes and are not open for college student activities, resulting in little impact on college students’ behavioral patterns and psychological restoration.

### 5.3. Influence paths and effects of the characteristics of rest facilities

**Figure 9:**
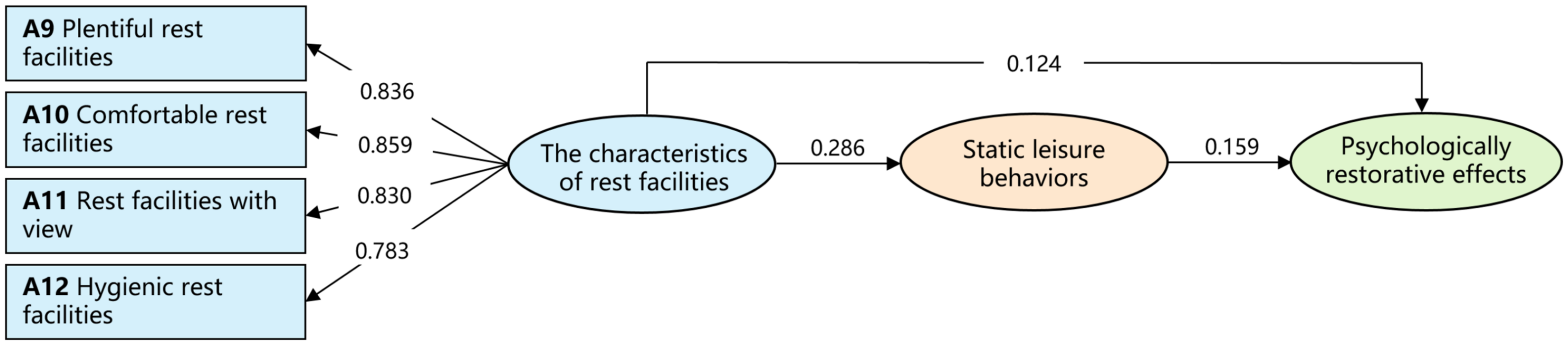
Influence effect illustration of the characteristics of rest facilities.

According to the research results, the total effect size of the characteristics of rest facilities to psychological restorative effects is 0.169 (Table 4), accounting for 16% of the total effects. It can be seen from Figure 9 that the influence effect of the characteristics of rest facilities on static leisure behaviors is 0.286. Among the path coefficients of the four measurement indicators of the characteristics of rest facilities, “A10 Comfortable rest facilities” (0.859) represents the most important influencing factor. It can be inferred that in campus common spaces, the comfort level of rest facilities has the highest impact on college students’ static leisure behaviors and psychological restoration needs. College students with a high demand for psychological restoration are more concerned about the comfort level of rest facilities in relaxing themselves, so comfortable seats can induce more static leisure behaviors. The quantity, location and orientation of rest facilities can create more opportunities for college students to rest and spend more time in campus common spaces. In addition, college students with psychological fatigue are more sensitive to environmental hygiene. Clean and tidy seats in campus common spaces can, to a certain extent, promote college students’ static leisure behaviors.

### 5.4. Influence paths and effects of the characteristics of activity facilities

**Figure 10:**
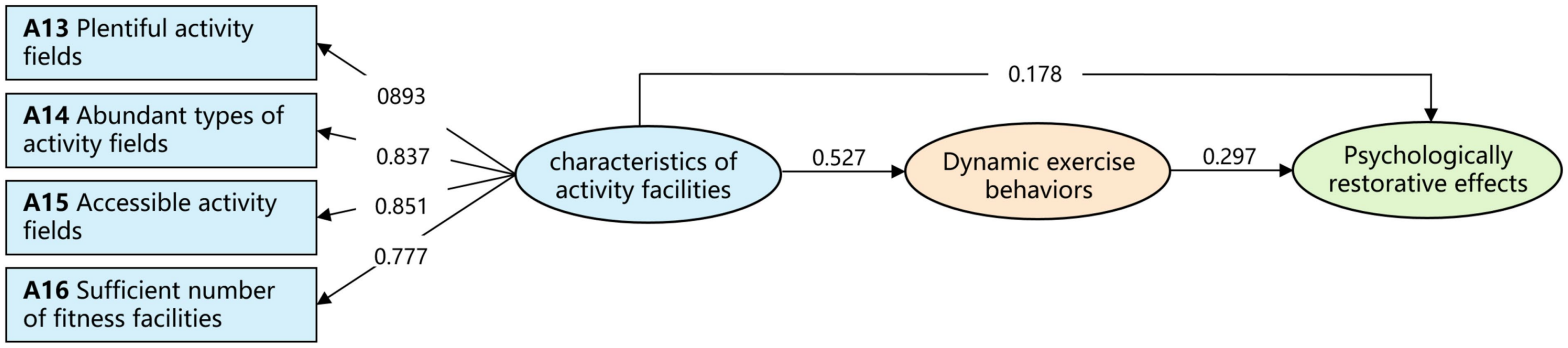
Influence effect illustration of the characteristics of activity facilities.

The characteristics of activity facilities mainly describe the configuration of sports fields and fitness facilities on campus. The research results show that the effect size of the characteristics of activity facilities to psychological restorative effects is 0.334 (Table 4), accounting for 33% of the total effects. Through the comparison of path coefficients in Figure 10, it can be seen that the influence effect of the characteristics of activity facilities on dynamic exercise behaviors is 0.527, the greatest impact compared with other characteristics of common spaces. Among the path coefficients of the four measurement indicators of the characteristics of activity facilities, “A13 Plentiful activity fields” (0.893) represents the most important influencing factor. It reveals that, compared with fixed fitness facilities, college students prefer flexible activity fields, which have a higher impact on their dynamic exercise behaviors. This is consistent with the research results of Yu et al., i.e., a sufficient number of sports fields on campus can promote college students’ dynamic exercise behaviors (44). Some studies on community common spaces have shown that fitness facilities in common spaces can better promote dynamic exercise behaviors than activity fields (64). This discrepancy is mainly due to different research groups. The majority of community residents are middle-aged and older adults, who prefer mild or moderate fitness activities, while college students prefer more competitive, flexible and collaborative field activities. In addition, the accessibility of activity fields can increase the frequency of college students’ exercise activities, while a great variety of them can improve the college students’ choices and the duration of exercise activities, both playing a significant role in promoting psychological restoration.

### 5.5. Limitations in the research

Overall, the research reveals how the characteristics of campus common spaces promote the psychological restoration of college students. In particular, differing from existing studies, the research confirmed the direct and indirect effects of the characteristics of campus common spaces on psychological restoration, using college students’ behavioral patterns as mediating variables. Nevertheless, some limitations were noted during the research, which require further improvement. First, the structural equation model constructed in the research needs further improvement. The observed variables for the characteristics of campus common spaces mainly extracted physical environment factors and did not cover perceived environmental factors, such as the security and privacy of the space. Future research should explore these factors that were not covered here. In addition, it is necessary to better identify the influence of campus common spaces’ characteristics on the psychologically restorative effects on college students. It is important to explore more mediating factors, such as perceptual restorative and emotional responses, in future research. Second, the number of samples and the scope of the research were limited, so the universality of the research conclusions needs to be further verified; in addition, different campus environments have different impacts on college students, so the future research will further expand the scope of samples and conduct a comparative study of different types of campus common spaces. Finally, due to the limitations of the practical operating conditions, the research used mainly short questionnaires to measure college students’ psychological restoration; in the future, biosensor technology should be employed to collect college students’ ECG, EEG, ECOG, EMG and other autonomic nerve response data in campus common spaces, and the research should explore the rules regarding the impact exerted by the environmental characteristics of campus common spaces on college students’ psychological restoration from both qualitative and quantitative aspects.

## 6. Conclusion

### 6.1. Main conclusion and innovation points

From the perspective of college students’ psychological health, the author constructed a theoretical model for the psychologically restorative effects of campus common spaces based on a field survey of 22 common spaces in the SCUT Wushan Campus, as well as a follow-up analysis of spatial characteristics and the extraction of behavioral activities. Structural equation modeling (SEM) was adopted to test the theoretical model, with an analysis of the paths and effects by which campus common spaces influence the psychologically restorative effects upon college students. The main conclusions are as follows. First, the research confirms that the four characteristics of campus common spaces all have psychologically restorative effects. Among them, the characteristics of activity facilities and the landscape environment have the greatest impact on psychologically restorative effects; they are followed by those of the architectural environment; those of rest facilities have the least impact. Together, they constitute the four dimensions of the restorative environment of campus common spaces. Second, the research confirms that the environmental characteristics of campus common spaces not only directly affect the psychological restoration of college students but also produce psychologically restorative effects through the interaction between the characteristics of campus common spaces and college students’ behavioral patterns. The mediation effect of college students’ behavioral patterns accounts for around 41% of the total effects of psychological restoration, a relatively high proportion, in which the psychologically restorative effect of dynamic exercise behaviors is 2.5 times that of static leisure behaviors. Therefore, the psychologically restorative effects of campus common spaces are closely related to the behaviors of college students in common spaces. The design of a restorative campus environment should focus on not only spatial characteristics but also on the behavioral patterns of college students.

The research puts forward the viewpoint of addressing the psychological health problems of college students by guiding their behaviors through the characteristics of campus common spaces, identifies the correlation between the environmental characteristics of spaces and psychologically restorative effects and realizes the quantifiable research of non-quantifiable factors through the construction of a structural equation model (SEM) for the psychologically restorative effects of campus common spaces from the perspective of college students’ behavioral patterns. It effectively promotes related research on restorative environments on campus, and it provides new ideas for the construction of a healthy campus. The research results can guide the optimization of the design of campus common spaces and provide a theoretical basis and workable measures for the construction of campus common spaces that can meet the psychological health needs of college students.

### 6.2. Inspirations

As an integral part of and an important restorative place on a college campus, common spaces serve as an important form for the daily lives and communication of college students. Therefore, after identifying the psychological restoration mechanism of campus common spaces based on college students’ behavioral patterns, the author puts forward recommendations for the design of campus common spaces from three aspects, i.e., architectural design, landscape design and facility configuration as per the “static leisure” and “dynamic exercise” behavioral patterns. The objective is to establish a health support mechanism for college students who suffer from psychological depletion, such as attention fatigue, stress and negative emotions.

#### 6.2.1. Improve the diversity of building enclosures in common spaces

From the research results, it can be seen that the characteristics of the architectural environment in campus common spaces have an important impact on the dynamic exercise behaviors of and psychologically restorative effects upon college students. Within the category of the said characteristics, the building enclosure scale, form and cultural atmosphere are the crucial factors. Therefore, the design of campus common spaces should create multiple types of building enclosure spaces supporting behavioral activities through the building enclosure scale and form. For example, common spaces such as campus squares and courtyards may be divided into various functional areas. The resulting diversified types of common spaces can provide college students with a rational place to engage in public activities, promote their behavioral activities and thus facilitate psychological restoration. In addition, the design of campus common spaces should integrate the regional context and campus historical and cultural elements into architectural interfaces so as to enrich the cultural atmosphere of the common spaces, enable college students to perceive different spaces through characteristics of historical context, enhance their cultural and aesthetic experience in common spaces and thus promote their psychological restoration.

#### 6.2.2. Enhance the full sensory experience of the landscape environment in common spaces

This paper proves that the characteristics of the landscape environment have a great impact on the static leisure behavioral patterns of and psychologically restorative effects upon college students. In the design of campus common spaces, the visual, auditory and tactile experience brought about by the landscape environment should be enhanced to induce college students’ leisure behaviors and thus promote their psychological restoration. First, the enhancement of visual experience is necessary. Plant type and color are the landscape design elements that promote college students’ visual experience. In landscape design, different colors, shapes and uses of plants should be leveraged and plant communities should be reasonably configured to foster a comfortable, pleasant spatial environment. Second, the enhancement of auditory experience is necessary. The design of campus common spaces should also focus on waterscapes by, for instance, creating visually and auditorily pleasing living water landscapes (such as cascades, fountains, and streams). Third, the enhancement of the tactile experience is necessary, by creating a landscape environment conducive to college students’ active participation. For example, as many large lawns are present in the form of inaccessible green landscapes with a low utilization rate, lawns on campuses should be preferably opened up and integrated with the pedestrian road network to allow college students to sit on them and interact with others while enjoying a pleasant connection with nature.

#### 6.2.3. Optimize the compatibility between facility configuration and behavioral activities in common spaces

This study indicates that the characteristics of activity facilities in campus common spaces have the greatest impact on the dynamic exercise activities of and psychologically restorative effects upon college students, with the quantity and accessibility of activity fields being the crucial factors. However, in previous campus designs, sports fields were either insufficient due to a shortage of land or far away from teaching and living areas, thus being less utilized or simply abandoned. Therefore, in the design of university campuses, it is necessary to not only provide a sports field that meets the basic exercise needs of students, but also create varied, continuous activity spaces in the front and back yards of buildings or idle spaces. It is also necessary to reasonably plan the service radius and spatial distribution of facilities for high-intensity sports (such as basketball courts, tennis courts and track and field) in the living areas of college students, and design facilities for low-intensity sports (such as badminton courts) on small squares and courtyards in the teaching area. In this way, more opportunities for diversified physical activities are made available for college students. In addition, the characteristics of rest facilities in common spaces have a certain impact on the static leisure behaviors of and psychologically restorative effect upon college students, with the comfort level and quantity of rest facilities being crucial factors. Therefore, in the configuration of rest facilities in campus common spaces, a proper number of comfortable rest facilities should be provided for college students to relax, read and rest, with a reasonable layout or movable seats to accommodate the college students’ needs for relaxation, meditation or gathering.

## Data availability statement

The original contributions presented in the study are included in the article/supplementary material, further inquiries can be directed to the corresponding author.

## Ethics statement

Ethical review and approval was not required for the study on human participants in accordance with the local legislation and institutional requirements. Written informed consent from the participants was not required to participate in this study in accordance with the national legislation and the institutional requirements.

## Author contributions

WG: conceptualization, resources, software, writing—original draft preparation, writing—review and editing. HW: conceptualization, methodology, software, validation, formal analysis, investigation, data curation, visualization, writing—original draft preparation, writing—review and editing. XL: conceptualization, methodology, visualization, software, writing—original draft preparation, writing—review and editing, funding acquisition. All authors contributed to the article and approved the submitted version.

## Funding

This research is supported by the 2022 Guangdong Philosophy and Social Science Foundation (grant no. GD22XGL02); the Fundamental Research Funds for the Central Universities (grant no. QNMS202211); the National Natural Science Foundation of China (grant no. 52108011); Guangdong Basic and Applied Basic Research Foundation (grant no. 2023A1515011137); Guangzhou Philosophy and Social Science Planning 2022 Annual Project (grant no. 2022GZQN14); Department of Education of Guangdong Province (grant no. 2021KTSCX004); Department of Housing and Urban–Rural Development of Guangdong Province (grant no. 2021-K2-305243); Science and Technology Program of Guangzhou, China (grant no. 202102020302); State Key Laboratory of Subtropical Building Science, South China University of Technology (grant no. 2021ZB16); and China Postdoctoral Science Foundation (grant no. 2021 M701249).

## Conflict of interest

WG and XL were employed by Architectural Design and Research Institute Co., Ltd.

The remaining author declares that the research was conducted in the absence of any commercial or financial relationships that could be construed as a potential conflict of interest.

## Publisher’s note

All claims expressed in this article are solely those of the authors and do not necessarily represent those of their affiliated organizations, or those of the publisher, the editors and the reviewers. Any product that may be evaluated in this article, or claim that may be made by its manufacturer, is not guaranteed or endorsed by the publisher.

## Appendix

APPENDIX 1 Goodness-of-fit test of the structural equation model.

**Table:**

Fit indices	Reference value	Model value	Overall model fit
**χ**^**2**^		558.151	
df		280	
**χ**^2^**/**df	<2.00	1.993	Yes
GFI	>0.90	0.921	Yes
AGFI	>0.90	0.901	Yes
PGFI	>0.50	0.735	Yes
RMR	<0.05	0.016	Yes
RMSEA	<0.05	0.046	Yes
CFI	>0.90	0.972	Yes
NFI	>0.90	0.946	Yes
RFI	>0.90	0.937	Yes
IFI	>0.90	0.972	Yes
TLI	>0.90	0.968	Yes

APPENDIX 2 Test of path model.
